# Cyber and Offline Dating Abuse in a Portuguese Sample: Prevalence and Context of Abuse

**DOI:** 10.3390/bs10100152

**Published:** 2020-10-05

**Authors:** Sónia Caridade, Hélder Fernando Pedrosa e Sousa, Maria Alzira Pimenta Dinis

**Affiliations:** 1 Faculty of Human and Social Sciences, University Fernando Pessoa (UFP), Praça 9 de Abril 349, 4249-004 Porto, Portugal; 2 Permanent Observatory Violence and Crime (OPVC), University Fernando Pessoa (UFP), Praça 9 de Abril 349, 4249-004 Porto, Portugal; madinis@ufp.edu.pt; 3 Interdisciplinary Center for Gender Studies (CIEG) of the Higher Institute of Social and Political Sciences, University of Lisbon (ISCSP-UL), 1300-663 Lisboa, Portugal; 4 Department of Mathematics (DM.UTAD), University of Trás-os-Montes and Alto Douro (UTAD), Quinta de Prados, 5001-801 Vila Real, Portugal; hfps@utad.pt; 5 UFP Energy, Environment and Health Research Unit (FP-ENAS), University Fernando Pessoa (UFP), Praça 9 de Abril 349, 4249-004 Porto, Portugal

**Keywords:** cyber dating abuse (CDA), offline dating abuse (ODA), information and communication technologies (ICT), dating relationships, prevalence, context of dating abuse

## Abstract

The increasing use of information and communication technologies (ICT) and networking has promoted the occurrence of different forms of victimization, specifically in terms of interpersonal interaction (e.g., cyberbullying or online risk-taking behaviour), which also includes cyber dating abuse (CDA). Some studies report that CDA is an extension of offline dating abuse (ODA). Because Portuguese studies in this area are scarce, this study aims to bridge this knowledge gap, seeking to investigate the extent and the context of CDA occurrence, and the relationship between CDA and all forms of ODA, i.e., physical, verbal–emotional and control. A convenience sample of 173 Portuguese adolescents and young adults was studied. This sample is mostly female (86.7%), has a mean (M) age of 25.36 years old and a standard deviation (SD) of 6.88 years of age. The results show that CDA and ODA among the Portuguese are very prevalent and related. Control (31.8 vs. 20.8%) and verbal–emotional ODA (26.7 vs. 20.2%) as well as control CDA (38.2 vs. 43.4%) were the most prevalent forms of abuse, either in terms of victimization or in terms of perpetration. The results also showed that CDA usually appears in a context of jealousy, also explaining control CDA and CDA direct aggression. A significant relationship between control CDA and CDA direct aggression and physical, verbal–emotional and control ODA was found. Experiencing control and verbal–emotional ODA are the main risk factors of control CDA victimization. This study allows to lay the groundwork for further research on dating victimization and perpetration through ICT, and has important practical implications at the level of Portuguese prevention strategies and intervention policies, thus emphasizing the role of the official authorities and the law itself.

## 1. Introduction

The use of communication and information technologies (ICT) is currently part of the daily routine of many adolescents and young adults. The wide variety of digital and technological tools currently available, such as text messages, numerous social networks (e.g., Facebook, Instagram, Tumblr or Twitter), email, mobile phones and the use of a webcam, among others, allows young people to easily establish an online dating relationship [1], and is also a simple way to be connected with the loving partner, aiming to express affection [2]. However, digital practices and extensive network use also trigger additional problems associated with the disinformation about the risk of using these specific tools. The research on the relevance that ICT may have in promoting violence and cyber violence has greatly increased in recent years worldwide. It has been reported that ICT contribute to making adolescents and young people more susceptible to interpersonal intrusiveness, thus endorsing different forms of victimization such as cyberstalking [3], cyberbullying [4], sexting [5] and cyber dating abuse (CDA) [1,6,7], among other forms of online victimization.

CDA is one form of victimization practiced through ICT use, consisting of a form of control and harassment by the dating partner [8]. Considered a multidimensional construct, CDA can involve multiple abusive behaviours through digital practices. Examples are daily control and surveillance of the dating or ex-dating partner; sending/posting offensive or humiliating comments to/of the dating partner; sending emails or messages containing different threats; and/or posting photos [1,9]—thus integrating different abusive typologies. The literature reports the existence of a continuous effort to establish CDA abusive typologies, although with little consensus. The review developed by Gámez-Guadix et al. [10] identified the following typologies: cyber control or monitoring (e.g., the need to know, at all times, the whereabouts of the dating partner and where he/she is); cyber harassment (e.g., repeated and insidious calls); and cybernetic and psychological aggression (e.g., insults, threats and humiliations). Other authors, such as Watkins et al. [11], outlined different CDA typologies (e.g., psychological cyber aggression, sexual cyber aggression and cyber aggression). CDA direct aggression, which includes behaviours to harm victims through direct attacks (e.g., threats, insults or dissemination of private information), and control CDA, as a means of cybernetically monitoring social relationships and the entire behaviour of victims, constitute two other cited recurrent typologies [12,13,14]. The many studies [7,14,15,16,17] focusing on these two types of abuse report a greater preponderance of CDA control behaviours in relation to partners, when compared to CDA direct aggression behaviours.

Despite the high variability in prevalence rates, worrying percentages of CDA victimization and perpetration by young people have been reported. A systematic review of 44 studies on young CDA victims, carried out by Caridade et al. [18], found the minimum rates of victimization and perpetration to be around 6% and the maximum rates of victimization and perpetration exceeding 90% (92 and 93.7%, respectively). The critical review of CDA measures by Brown and Hegarty [19] found perpetration rates among youth ranging from 6 to 91%. Another review of 21 studies investigating digital dating abuse, made by Stonard et al. [20], found victimization and perpetration rates of around 55%, also showing that victimization ranged from 12 to 56% and perpetration ranged from 12 to 54%. Peskin et al. [21] also found that 15% of the participants involved in their study had already perpetrated some kind of abuse throughout their lives, using the social networks. The studied research, e.g., [18,20,22] allows to conclude that CDA investigations have produced results that may be considered as conflicting and which are also difficult to interpret. This can be attributed to the lack of consensus in relation to the terminology used, the operationalization of variables and the tools used to measure CDA, as well as to the methodological characteristics of the studies (e.g., sample size, sampling context, selection method or time interval considered) [10,18,19,20,22]. The literature has also documented the existence of CDA reciprocity. Associations between CDA victimization and perpetration have been reported by several studies investigating reciprocity, e.g., [11,14,23,24], and this reciprocity also happens in traditional dating violence [25].

Considering the context in which dating violence occurs, it is essential to better understand the meaning and motivation behind this type of abuse, as well as to design effective preventive strategies and intervention policies at the institutional level. International research has documented that many abusive behaviours in traditional dating relationships [26] involving ICT use [27,28] are attributed to a context of “play” or “joking”. The studies that document a higher prevalence of control CDA [16,17] have also reported that the belief in romantic dating myths among adolescents is behind this type of abuse. Jealousy, commonly perceived as a sign of love in affective relationships, is an example of that. Other studies [29,30] have also identified the partner’s jealousy and anger as important explanatory elements in the context of the partner’s intimate aggression. In the study developed by Rodríguez-Domínguez et al. [28], comprising a sample of 206 Spanish high school students, it was also found that the attitudes and beliefs based on sexism and jealousy motivated the occurrence of early dating violence, thus transcending the virtual space. This evidence was also corroborated in a Portuguese study carried out by Santos and Caridade [31], where jealousy as well as individual and partner self-control abilities (impulsivity/aggressiveness) were the most mentioned indicators by the participants, as the main causes of dating violence.

CDA could be considered as an extension of Offline Dating Abuse (ODA), something that has motivated several empirical studies to investigate the relationship between these types of abuse, e.g., [7,12,16,17]. The main focus of this study is the CDA and ODA relationship in the Portuguese context, contextualized at an international level. There are no studies investigating the association between CDA and ODA among adolescents or adults in dating relationships in Portugal.

### 1.1. Cyber and Offline Dating Abuse Relationship

ODA is a prevalent, widespread and public health problem. Several international, e.g., [25,32,33,34] and Portuguese, e.g., [31,35,36,37] studies have been documenting the high rates of dating violence and the consequences it has for victims. Defined as an aggressive behaviour perpetrated by the boyfriend/girlfriend against her/his dating partner [32], ODA could involve the use or threat of different typologies of violence, either physical, emotional/psychological or sexual [38], in relation to both heterosexual and homosexual [39] partners. The Center for Disease Control and Prevention [40] in the United States (US) reports that one in five girls and one in seven boys have suffered some form of intimate partner violence between 11 and 17 years of age. The higher rate of victimization between women is also reported in a systematic review by Jennings et al. [34], comprising 169 studies and involving young people aged 15 to 30 years old. A lower prevalence estimates among younger (<10%), when compared to older people (between 20% and 30%), was also demonstrated in the same structured review.

As is happening in other European countries (e.g., France, Germany or Spain) [33], Portuguese studies have documented high rates of dating violence among adolescents and young people, e.g., [35,36,37]. In a Portuguese study by Guerreiro et al. [41], carried out with a sample of 2500 young people aged 12 to 18 years, 7% of the participants acknowledged having suffered ODA at least once, and psychological violence emerged as the most prevalent form of violence (8.5%), followed by physical violence (5%) and by sexual violence (4.5%). Higher rates of ODA were found in another study carried out in the Portuguese context by the Union and Response of the Alternative Woman (UMAR) [42], with a sample of 4938 young people from all districts of Portugal. In this same study, 58% of the young people studied admitted having suffered at least one form of violence and 67% of the total of young people inquired legitimized at least one behaviour of violence. Although there are other abusive behaviours in intimate interactions, such as relational, verbal–emotional [16], controlling and fear/intimidation behaviours [43], it may be stated that physical, psychological and sexual violence are the three types of abuse that are most commonly reported in the studied research, e.g., [32,35,36]. In the context of dating relationships, adolescents and young adults tend to experience and perpetrate more than one type of violence (e.g., psychological and sexual violence) in the same or different situations [44]. Experiencing these different forms of violence may also be associated with ICT use [45].

International research has also been documenting the co-occurrence and a positive relationship between CDA and ODA, arguing that CDA may constitute a form of psychological violence in dating [12,20,28,46,47,48,49]. CDA also has been associated to other forms of violence, such as cyberbullying [3,12] or bullying [50]. A study by Zweig et al. [49], using a sample of 3745 youths in the north-eastern US, concluded that CDA often co-occurs with other forms of dating violence, in particular with sexual violence. In the same study, half of the victims of sexual CDA were also victims of physical violence, and nearly all were victims of both types of CDA, i.e., victims of sexual and physical violence also experienced other abusive psychological experiences. In a study comprising a sample of 433 Spanish college students, aged 18 to 30 years, a significant relationship between CDA and psychological ODA was found by Borrajo et al. [12]. In Spain, another study by Cava et al. [16], involving a sample of 492 adolescents, also corroborated the association between ODA and CDA victimization. Specifically, ODA victimization emerged as a significant risk factor of victimization by control CDA and CDA direct aggression, for boys and girls. ODA victimization by verbal and emotional violence emerged as the main risk factor of victimization by control CDA. On the other hand, CDA victimization by physical and relational violence emerged as the main explanatory variables of victimization by CDA direct aggression [16].

CDA and ODA are very relevant and prevalent social problems and, while still to be related to each other, have characteristics that differentiate them and justify a greater investment in this area, namely in terms of research and consequent intervention at the practical level. It is extremely difficult for a victim to escape the online abuse. It is known that, in the case of online abuse, the probability of repeated victimization is higher, since some offenders may suffer less inhibition against that specific form of abuse [51].

### 1.2. The Present Study

Despite the numerous Portuguese studies on ODA in the Portuguese context, e.g., [35,36,37], studies on the CDA prevalence, e.g., [15], and studies testing the association between CDA and ODA, e.g., [7], are scarce. This study aims to fill a gap in the Portuguese literature in this field, to contribute to the increasing knowledge about the relationship between CDA and ODA in a Portuguese convenience sample. The four objectives of this study are (i) to determine the extent to which participants used ICT (e.g., messages, email and social networks) to interact with partners in dating relationships; (ii) to investigate the prevalence of CDA (i.e., control and direct aggression) and ODA (i.e., physical, verbal–emotional and control), in terms of victimization and perpetration patterns; (iii) to determine the context in which CDA occurs (e.g., jealousy, infidelity, joking or reciprocity); and (iv) to investigate whether ODA is correlated with CDA, as well as to determine the role of ODA victimization and perpetration (i.e., physical, verbal-emotional and control) as explanatory variables of CDA victimization and perpetration (i.e., control and direct aggression). Regarding the last objective (iv), the following two hypotheses were formulated:

**Hypothesis** **1.** *Positive correlations among all three forms of ODA (i.e., physical, verbal–emotional and control) and CDA (i.e., control and direct aggression) are expected in both victimization and perpetration patterns.*

**Hypothesis** **2.** *ODA victimization and perpetration (i.e., physical, verbal–emotional and control) are expected to be explanatory variables of CDA victimization and perpetration (i.e., control and direct aggression).*

## 2. Materials and Methods

### 2.1. Sample

This study included a convenience sample of 173 Portuguese adolescents and young adults, mostly female (86.7%), mainly college students (48.6%) and with a mean (M) age of 25.36 years and standard deviation (SD) of 6.88 years. A significant percentage of the participants were attending the 1st cycle of studies (48.6%), with 12.7% in the 2nd cycle of studies and the residual in postgraduate attendance (0.4%). A total of 32.4% of the participants reported other non-tertiary education. Regarding the affective relational situation, 75.1% of the participants revealed to have been involved in a dating relationship in the last year, with the remaining 24.9% reporting that the dating relationship was already over. The average duration of the dating relationship was 36.20 months (SD = 12.76 months) (Table 1).

### 2.2. Procedure

The questionnaires’ protocol was initially made available through an online survey on the Google Docs online platform. Then, the Uniform Resource Locator (URL) with the questionnaires was later disseminated by the institution in charge of this study through an informative announcement by e-mail, social networks (e.g., Facebook, Messenger or WhatsApp) and contacts established with different universities. Before asking the participants to complete the questionnaire, a brief description of the study was presented, clarifying the objectives, the inclusion criterion (i.e., having experienced dating relationships), the confidentiality and anonymity of the data, the voluntary nature of the responses and the absence of any compensatory economic participation, as well as the time to complete the questionnaires (about 20 min). The participants were also informed that they should answer only once. The informed consent was also made available, and the participants could only advance in the completion of the instruments after indicating (obligatory completion item) their agreement to participate in the study. The questionnaires were available online between October and November 2019, and the process was completed in December 2019. The study was conducted in accordance with the Declaration of Helsinki, and the protocol was approved by the Ethics Committee of University Fernando Pessoa (UFP) Porto, Portugal, Project “Relationship Between Cyber Abuse and Offline Violence in Juvenile Intimate Relationships”, in March 2019. No specific reference was assigned, with the date acting as the reference ID.

### 2.3. Measures/Instruments

Background Questionnaire: It included a series of questions about age, sex, whether the participant has or had a relationship and its duration in months.

Types of ICT Used: Participants were asked about the type of ICT used to interact with the intimate partner, and the frequency with which they did it. More specifically, participants had to indicate whether they had ever used the following digital tools and the frequency with which they did so through a 5-point Likert scale (“I have never used it” until “I always use it”): messages, e-mail, WhatsApp, Facebook, Twitter, Instagram and Tumblr.

Context of Abuse: Participants were asked to mention the context in which the dating violence had occurred. Specifically, they had to indicate one or more of the following contexts: jealousy; angry; infidelity; “game” or “joke”; a context of reciprocity (i.e., the aggressor did it first); or other.

Offline Abusive Dating Experiences Questionnaire [31]: An adapted version of this instrument was used in the present study. It considered only Section 4 on the characterization of abusive dating experiences. Three forms of ODA victimization and perpetration (i.e., physical, verbal–emotional and control) were measured. The physical abuse subscale comprises six items, describing situations in which the participants have experienced and/or perpetrated physical abuse by/to their partners, such as being pushed or hit, kicks, throwing objects, pushing (e.g., “*My partner slapped me or push me*”). The verbal–emotional abuse subscale includes four items, describing situations in which the participants have experienced/perpetrated verbal/emotional abuse by/to their partners, such as insults, threats and humiliation (e.g., “*My partner insulted me with put-downs*”). The control abuse subscale includes six items, contemplating situations in which individuals report that their partners control their behaviours and observe their movements, such as stopping to talk or going out with friends, researching on the phone their behaviours and watching their movements, such as stopping to talk or hanging out with friends, or searching the phone (e.g., “*My partner doesn’t allow me to go out or talk to friends*”). The participants responded to these items with three options: 0 (never), 1 (once), 2 (more than once). Cronbach’s alpha (α) was measured, and reliable values for the victimization and perpetration of the different types of abuse, namely, physical abuse (α = 0.70 and α = 0.72, respectively), verbal–emotional abuse (α = 0.88 and α = 0.70, respectively) and control abuse (α = 0.83 and α = 0.71, respectively), were obtained.

The Cyber Dating Abuse Questionnaire (CDAQ) [21] was translated and validated for the Portuguese population by Caridade and Braga (CibAN—Cyber Abuse in Dating) [15] to assess CDA occurring within the scope of dating relationships, and investigating reciprocity in terms of victimization and perpetration. CibAN is a self-report measure, consisting of 40 items about various types of CDA, such as threats, identity theft, control and humiliation. The 20 items intended to assess the victimization indicator (e.g., “*My partner or former partner made a comment on a wall of a social network to insult or humiliate me*”) and the other 20 items made it possible to estimate the perpetration indicator (e.g., “*I wrote a comment on the wall of a social network to insult or humiliate my partner or former partner*”). The response scale used was a 6-point Likert scale: 1 (never); 2 (not in the last year, but before); 3 (once or twice); 4 (3 to 10 times); 5 (10 to 20 times); and 6 (more than 20 times). The questionnaire integrates two factors: Direct aggression (e.g., “*I threatened my partner or former partner using new technologies to physically hurt her/him*”) and control (e.g., “*Using mobile applications, I controlled the hour of the last connection with my partner or former partner*”). The reliability of the scale for this sample was *α* = 0.92 for victimization of direct aggression and *α* = 0.94 for victimization of control; *α* = 0.88 for perpetration of direct aggression and *α* = 0.87 for perpetration of control.

### 2.4. Data Analysis

The Statistical Program for Social Sciences was used for statistical analysis (IBM SPSS for Windows, version 26.0, IBM Corp, Armonk, NY, USA), including descriptive statistical analyses. The descriptive univariate analyses were computed to characterize the sample, to determine the extent to which the participants used ICT to interact with partners in dating relationships (Objective i); to investigate the prevalence of CDA and ODA victimization and perpetration and their abusive typologies (Objective ii); and to determine the context in which CDA occurs (e.g., jealousy, infidelity, joking or reciprocity) (Objective iii). Bivariate descriptive and inferential statistics were computed to investigate the relationship between CDA and ODA (Objective iv). Specifically, Pearson’s correlations among ODA (i.e., physical, verbal–emotional and control) and CDA (i.e., control and aggression CDA) were calculated, considering that the assumptions of normality and homogeneity were guaranteed.

Finally, linear regression analyses were carried out to investigate the role of ODA (i.e., physical, verbal–emotional and control) and jealousy as explanatory variables of control CDA and CDA direct aggression victimization and perpetration. Although the main goal was to explore different reasons for the occurrence of ODA (i.e., jealousy, infidelity, joking or reciprocity), only jealousy emerged as the main reason pointed out by the participants, which is why it was the single one included in the multilinear regression analysis. The associations between jealousy and different forms of ODA victimization and perpetration (i.e., physical, verbal–emotional and control), as possible explanatory variables of the different typologies of CDA (i.e., direct aggression and control) victimization and perpetration patterns, were assessed with separate multiple linear regression models. Thus, four different multilinear regression analyses were performed, using the stepwise method. This allows to obtain a model in which the different explanatory variables X perfectly explain the outcomes’ variables Y, as also suggested by Field [52].

## 3. Results

### 3.1. Descriptive Statistics

#### 3.1.1. Frequency of ICT Use

In order to better understand the extent of ICT use, the participants were asked about the frequency of their digital practice, with 99.4% assuming to use ICT to interact with their partners. The most frequent use was reported in relation to messages (50.9%) and Instagram (34.7%) followed by Facebook (23.7%), e-mail (23.7%) and WhatsApp (20.2%). The social networks Twitter (14.5%) and Tumblr (11%), less used in the Portuguese context, emerged as being the least used by the participants in this study.

#### 3.1.2. Cyber Dating Abuse: Prevalence and Context of Abuse

Of all the participants who stated having been engaged in dating relationships, 40.2% reported having experienced at least some type of CDA and 42.2% assumed to have perpetrated at least one act of CDA. ODA presented indicators that were equally prevalent and disturbing, with 39.9% of the participants reporting to have been subjected to some kind of abuse in their dating relationship and 30.1% acknowledging to use some form of ODA (Table 2). Moreover, 69.2% of the participants experienced both CDA and ODA and 53.8% of the participants perpetrated both CDA and ODA. Analysing the different typologies of CDA, most participants assumed to have suffered (38.2%) and practiced (43.4%) some type of intimate control through ICT use. With regard to direct aggression, lower prevalence indicators were found in terms of victimization (8.1%) and perpetration (15.6%), although still worrying.

In relation to the typologies of ODA, control abuse was also more prevalent in terms of both victimization (31.8%) and perpetration (20.8%). The victimization and perpetration by emotional (27.7 and 20.2%, respectively) and physical violence (15.6 and 10.4%, respectively) were two other forms of ODA assessed, which also registered worrying indicators.

Participants were also asked to specify the context in which the abuse had occurred. A total of 54.9% reported that their partners acted in a context of jealousy, 21.4% acknowledged the reciprocity (e.g., “*I did this because my partner did it first*”), 10.4% reported it to be due to anger, 6.4% justified it because they were playing or joking and 6.9% indicated infidelity.

### 3.2. Relationship between Cyber Dating Abuse and Offline Dating Abuse

Table 3 shows the correlations between the variables included in the study, in terms of victimization and perpetration. The results indicated significant positive correlations among all forms of CDA (i.e., control and direct aggression) and ODA victimization (i.e., physical, verbal–emotional and control) in both victimization and perpetration patterns. However, regarding the jealousy variable, a significant negative correlation was only found with the control ODA victimization.

### 3.3. Jealousy and All Forms of Offline Dating Abuse as Explaining Cyber Dating Abuse

Using the stepwise method, a multilinear regression analysis was performed, with jealousy and all forms of ODA victimization (i.e., physical, verbal–emotional and control) as explanatory variables, and control CDA, separately for victimization and perpetration, as the dependent variables. Using the same stepwise method, another multilinear regression analysis was carried out. It considered jealousy and all forms of ODA perpetration as explanatory variables, as well as control CDA, also separately for victimization and perpetration. These two sets of analyses are presented in Table 4 and only the regression models with the highest coefficient of determination and with significant variables included are shown in each case. Regarding the dependent variables, control CDA victimization and perpetration, and verbal–emotional and control ODA victimization, as independent variables, this multilinear regression model explained 47.3% of the variance for victimization, and only 16.1% of the variance for perpetration. For CDA victimization, verbal–emotional ODA victimization (*β* = 0.256; *p* = 0.033) and control ODA victimization (*β* = 0.493; *p* < 0.001) explained control CDA victimization. Only control ODA victimization (*β* = 0.399; *p* < 0.001) explained control CDA perpetration. Considering jealousy, with control and physical ODA perpetration as independent variables, the multilinear regression model explained 26.0% for control CDA victimization and 16.4% for control CDA perpetration, as dependent variables. For control CDA victimization, jealousy (*β* = −0.300; *p* < 0.001), control ODA perpetration (*β* = 0.424; *p* < 0.001) and physical ODA perpetration (*β* = −0.230; *p* = 0.011) emerged as significant explanatory variables. In turn, only control ODA perpetration (*β* = 0.405; *p* < 0.001) explained control CDA perpetration.

Considering the other abusive typology of CDA, i.e., direct aggression, a multilinear regression analysis was carried out, using the stepwise method, with jealousy and all forms of ODA victimization (i.e., physical, verbal–emotional and control) as explanatory variables, and CDA direct aggression, separately for victimization and perpetration, as the dependent variables. Another multilinear regression analysis, using the same stepwise method, and considering jealousy and all forms of ODA perpetration as explanatory variables, as well as CDA direct aggression, also separately for victimization and perpetration, was carried out. Table 5 presents the corresponding two sets of analyses. Once again, only the regression models with the highest coefficient of determination and with significant variables included are shown in each case. Regarding the dependent variables, ODA direct aggression victimization and perpetration, and jealousy and verbal–emotional ODA victimization as the independent variables, the proposed multilinear regression model explained 29.2% of the variance for victimization and 24.3% of the variance for perpetration. Jealousy (*β* = 0.222; *p* = 0.016) and verbal–emotional ODA victimization (*β* = 0.514; *p* < 0.001) explained CDA direct aggression perpetration, but only verbal–emotional ODA victimization (*β* = 0.541; *p* < 0.001) explained CDA direct aggression victimization. Finally, in the last regression model carried out, only control ODA perpetration explained CDA direct aggression victimization (*β* = 0.259; *p* = 0.008) and perpetration (*β* = 0.345; *p* < 0.001).

## 4. Discussion

The main purpose of the present study was to investigate the prevalence of CDA and ODA abusive typologies, the context in which CDA occurs and examine the relationship between CDA and ODA, topics that are still less studied in the Portuguese context. This study is expected to represent a relevant contribution to the understanding of this specific victimization phenomenon and to contribute to the development of further studies in this area, with relevant implications at the level of policies to be implemented. The participants acknowledged the greater use of ICT (99.4%) to communicate and relate to their dating partners, with a predominance of the use of messages (58.9%) and some social networks (e.g., Instagram). These results confirm that adolescents and young adults have access to different forms to search and share information, also corroborating the extent and importance that the digital practices have in their daily routines [1]. The results also highlight the benefits of maintaining a healthy relationship in terms of connection, which, in turn, may also be translated into a higher risk of experiencing and/or perpetrating some forms of online abuse, including CDA [16].

As also reported by other relevant international studies, e.g., [20,51,53], the present study found high indicators in terms of CDA victimization (38.2%) and perpetration (42.2%). These results are lower than those found in a Spanish study by Cava et al. [16], with 68.8% of teenage girls reporting some ODA and also lower than the results of another Portuguese study by Caridade and Braga [15] using the same instrument, which found 59.2% by CDA victimization and 66.9% by CDA perpetration. As found in the study developed by Borrajo et al. [12], also with the same instrument, victimization and aggression for control CDA were higher (38.2% vs. 43.4%) when compared to CDA victimization and direct aggression (8.1% vs. 15.6%). Once again, these indicators are lower than those found in the aforementioned Portuguese study by Caridade and Braga [15], with respect to both victimization and aggression by control (58.8% vs. 62.3%, respectively) and victimization and perpetration by direct aggression (18.0% vs. 14.7%). It is important to highlight that the indicators of victimization and perpetration of control CDA are consistent with other international studies, e.g., [1,49], reporting rates below 50%. The increase in control CDA, when compared to CDA direct aggression, may be explained by the fact that it is a cyber abuse typology that includes abusive behaviours (e.g., persistent messaging or partner surveillance), being less explicit and therefore more acceptable by young people. It is also perceived as a sign of love or jealousy, as reported by Francis and Pearson [54]. In the analysis of the context in which the CDA may occur, jealousy was identified by this study participants as the most prevalent context of CDA (54.9%), corroborating what was reported by Borrajo et al. [12]. In addition, it has been documented that ICT use and networking tends to trigger control and jealous behaviours [55].

High indicators of ODA are also found in the present study, in accordance with other Portuguese, e.g., [31,35,36,37,41] and international [24,34] studies on this subject. Control ODA and verbal–emotional ODA were the most reported abusive typologies, either in terms of perpetration (20.8 and 20.2%, respectively) or in terms of victimization (31.8 and 27.7%, respectively), also corroborating the results of other international, e.g., [29,53,56,57] and Portuguese, e.g., [31,37,41] studies. Nonetheless, the preponderance of these types of psychological or control forms of abuse are of concern and should not be considered a devaluation of the problem of dating violence context. The evidence of escalating violence must not be overlooked [35]. These results show the importance of better understanding the risk factors that may be at the origin of these abusive behaviours. As an example, in an exploratory study involving 189 participants, Deans and Bhogal [58] found that some psychological factors, such as hostility and behavioural jealousy, explained CDA perpetration, thus highlighting the importance to understand the nature of these abusive behaviours.

The results in this study showed positive correlations between all forms of ODA victimization and perpetration (i.e., physical, verbal–emotional and control) and the two forms of CDA (i.e., control and direct aggression) in both victimization and perpetration patterns. The existence of this close connection between ODA and CDA has also been found in several previous relevant international studies, e.g., [12,16,46,47,48,49,53,59]. In effect, the literature has been conceptualizing CDA either as a form of psychological abuse, frequently related to face-to-face intimate violence [60], or as an extension of ODA [61]. In this study, jealousy has a positive significant association with control ODA victimization and emerged as an explanatory variable of control CDA perpetration, explaining 26% of the variance. These results seem to demonstrate, once again, the role that certain myths about love, namely the distorted and unrealistic view of young people about jealousy as being essential and natural in a dating, may have in increasing the experiences of victimization and perpetration in intimate relationships [62].

Finally, different risk factors, with diverse weights, were found for the CDA typologies control CDA and CDA direct aggression, as previously found by Cava et al. [16] in a Spanish sample. Verbal–emotional and control ODA victimization were the main risk factors of control CDA victimization in this study, explaining 47% of the variance, followed by both control ODA and physical ODA perpetration, explaining 26% of the variance. With a lower weight (around 16%) are the victimization and perpetration by control ODA in explaining control CDA perpetration. Regarding CDA direct aggression, verbal–emotional ODA victimization is the main explanatory variable of control CDA victimization, with a variance of 29%, and jealousy and control ODA victimization are the main risk factors of control CDA perpetration, explaining 24% of the variance. A longitudinal study developed by Temple et al. [59] showed that the CDA experiences tend to coincide with ODA involvement, concluding that adolescents and young people who are victims in one context (e.g., physical-to-face abuse) present a higher risk of victimization in the other context (e.g., ICT use), with detrimental implications for the adolescents’ psychosocial adjustment and developmental pathways.

## 5. Conclusions

Given the importance and relevance that the use of ICT has in the daily lives of adolescents and young adults, it is of crucial importance to investigate the risk factors involved in dating violence. A significant percentage of Portuguese adolescents and young adults having experienced CDA and ODA was found in the convenience sample of 173 participants in this study. The types of abuse most reported by the participants were control and verbal–emotional ODA and control CDA.

This study represents an important contribution to the limited empirical evidence of CDA in the Portuguese context. The results provide some enlightenment of the relationship between ODA and CDA and could be of explicit importance to improve the design of prevention strategies and intervention policies in this context, particularly attending to the role of official authorities and specific legislation in this field. Accordingly, it becomes increasingly necessary to integrate ICT knowledge into dating violence prevention programs in schools, but also to integrate all dating violence-related issues into broader programs that aim to reduce all forms of cyber aggression at an official level, which may also involve public recreational spaces. This study can be viewed as an expected contribution so that the evidence found in relation to risk factors involved in dating relationships may increase the future research to be developed in this very specific victimization field. The demonstrated co-occurrence of CDA and ODA signals the importance of necessary additional strategies at the institutional level to encourage a more cautious use of ICT, aiming to prevent specific situations between dating partners that are able to trigger the reported abusive behaviours. The Portuguese prevention efforts should focus on promoting healthy relationships, either through face-to-face interactions or when ICT use is involved. The strategies to achieve that must necessarily involve all parts of the problem, and shared knowledge is a crucial component of it.

This study presents some limitations that should be considered to better understand the findings. This is a study in a small country, Portugal, using self-report and retrospective measures and with a relatively small sample size and a mostly female participation (86.7%), which makes it difficult to generalize the results to the entire Portuguese population and may also have constrained the results in terms of multilinear regression analysis. The research methodology required time commitments from the participants and, therefore, limited the sample size, making the generalization of the results difficult. Consequently, and based on the results obtained, future research in this field requires further exploration of methods for practical application, such as regression including co-variates (non-stepwise model), involving a larger sample size of participants, sex balance and sample representativeness. In addition, the cross-sectional design used does not allow to clearly identify if ODA is a risk factor or a consequence of CDA. Aiming to clarify whether CDA is a distinct form of abuse, or if it constitutes an extension of ODA, as well as to investigate other variables that could be related to CDA (e.g., bullying and cyberbullying), additional longitudinal research is needed in the Portuguese context. Finally, it is also important to identify and understand the effects of ODA versus CDA to better comprehend its potential and possible differential impact, or if the cumulative abuse of both CDA and ODA results in a greater harm to the victim.

## Figures and Tables

**Table 1:** Participants’ characteristics (*n* = 173).

Variables	*n* (%)
Sex	
Female	150 (86.7)
Male	23 (13.3)
Education	
1st cycle of studies	84 (48.6)
2nd cycle of studies	22 (12.7)
Postgraduate	7 (0.4)
Other (non-tertiary education)	56 (32.4)
Relations situation	
In a dating relationship in last year	130 (75.1)
Dating relationship already over	43 (24.9)
	*M* (*SD*) (month)
Relationship duration	36.2 (12.8)

**Table 2:** Prevalence of cyber dating abuse (CDA), offline dating abuse (ODA) and related typologies (*n* = 173).

	Abusive Typologies	Victimization	Perpetration
		*n* (%)	*n* (%)
**CDA**	Control	66 (38.2)	75 (43.4)
	Direct Aggression	14 (8.1)	27 (15.6)
**ODA**	Physical	27 (15.6)	18 (10.4)
	Verbal–Emotional	48 (27.7)	35 (20.2)
	Control	55 (31.8)	36 (20.8)

**Table 3:** Pearson’s correlations among the variables of the study.

Variables	1	2	3	4	5	6
1. Jealousy		0.132	0.179	0.134	0.144	0.180
2. Physical ODA-V/P	0.175		0.393 *	0.431 *	−0.006	0.179 *
3. Verbal–Emotional ODA-V/P	0.142	0.515 **		0.520 *	0.178 *	0.294 **
4. Control ODA-V/P	−0.193 *	0.389 **	0.602 **		0.272 *	0.455 **
5. Direct Aggression CDA-V/P	0.018	0.221 **	0.352 **	0.350 **		0.205 *
6. Control CDA-V/P	0.133	0.222 **	0.431 **	0.499 **	0.335 **	

Note: The number of participants available for each correlation ranged from 156 to 172. V—Victimization; P—Perpetration; * *p* < 0.05; ** *p* < 0.01.

**Table 4:** Multilinear regression analysis assessing the control CDA victimization and perpetration associations.

Variable	Victimization ^1^			Perpetration ^2^		
	*R* ^2^	*β*	*t*	*R* ^2^	*β*	*t*
Verbal–Emotional ODA-V	0.473 ***	0.256	2.761 *	____	____	____
Control ODA-V		0.493	5.136 ***	0.161 **	0.399	4.443 ***
	**Victimization ^3^**			**Perpetration ^4^**		
Jealousy	0.260 ***	0.300	3.554 ***	____	____	____
Control ODA-P		0.424	4.784 ***	0.164 ***	0.405	4.514 ***
Physical ODA-P		−0.230	−2.573 *	_____	_____	_____

Note: *R*^2^: determination coefficient; *β*—standardized beta coefficient; *t*: Student’s *t*-distribution. ^1^ *F* (2, 107) = 48.082, *p* < 0.001, Adjusted *R*^2^ = 0.463; ^2^ *F* (1, 104) = 19.739, *p* < 0.001, Adjusted *R*^2^ = 0.151; ^3^ *F* (3, 106) = 12.412, *p* < 0.001, Adjusted *R*^2^ = 0.239; ^4^ *F* (1, 104) = 20.373, *p* < 0.001, Adjusted *R*^2^ = 0.156. * *p* < 0.05; ** *p* < 0.01; *** *p* < 0.001. V—Victimization; P—Perpetration.

**Table 5:** Multilinear regression analysis assessing the CDA direct aggression victimization and perpetration associations.

Variable	Victimization ^1^			Perpetration ^2^		
	*R* ^2^	*β*	*t*	*R* ^2^	*β*	*t*
Jealousy	____	____	____	0.243 ***	0.222	2.456 *
Verbal–Emotional ODA-V	0.292 ***	0.541	6.586 ***	0.243 ***	0.514	5.698 ***
	**Victimization ^3^**			**Perpetration ^4^**		
Control ODA-P	0.067 **	0.259	2.687 **	0.119 **	0.345	3.754 ***

Note: *R*^2^: determination coefficient; *β*—standardized beta coefficient; *t*: Student’s *t*-distribution. ^1^ *F* (1, 105) = 743.380, *p* < 0.001, Adjusted *R*^2^ = 0.286; ^2^ *F* (2, 103) = 16.495, *p* < 0.001, Adjusted *R*^2^ = 0.228; ^3^ *F* (1, 105) = 7.221, *p* = 0.008, Adjusted *R*^2^ = 0.061; ^4^ *F* (1, 104) = 14.089, *p* < 0.001, Adjusted *R*^2^ = 0.111. * *p* < 0.05; ** *p* < 0.01; *** *p* < 0.001. V—Victimization; P—Perpetration.
